# Subcutaneous foslevodopa/foscarbidopa initiation in a Parkinson’s day-clinic - a suitable setting to ensure treatment efficacy, tolerability and psychosocial adaption

**DOI:** 10.3389/fnagi.2025.1619850

**Published:** 2025-09-10

**Authors:** Alina Jander, Sarah Bergner, Beate Schönwald, Monika Pötter-Nerger, Carsten Buhmann, Ute Hidding

**Affiliations:** Department of Neurology, University Medical Center Hamburg-Eppendorf, Hamburg, Germany

**Keywords:** Parkinson’s disease, day-clinic, foslevodopa, motor fluctuations, quality of live, dyskinesias

## Abstract

**Background:**

Subcutaneous foslevodopa/foscarbidopa (LDp/CDp) has expanded the treatment options in advanced Parkinson’s disease (aPD). However, the most appropriate therapeutic setting for therapy implementation is not clear.

**Objective:**

To present a concept for LDp/CDp therapy implementation in a Parkinson’s day-clinic and efficacy and safety outcome data from patients under the new therapeutic regimen.

**Methods:**

Retrospective clinical data were collected from the first 24 patients with aPD who were initiated on LDp/CDp treatment at the Hamburg Parkinson’s day-clinic. Outcome parameters were analyzed in terms of motor symptoms (MDS -UPDRS II-IV), safety aspects and effects on patients’ quality of life (PDQ-39).

**Results:**

The concept of the Parkinson’s Day-clinic enabled the successful implementation of LDp/CDp therapy in patients with advanced Parkinson’s disease (aPD). It provided individualized medical supported via neurologists, specialized nurses and therapists and thus facilitated the transition from clinic-based care to home-based support. Compared to previous optimized oral treatment, the application of LDp/CDp significantly reduced motor complications such as dyskinesias and motor fluctuations by 53% on the MDS-UPDRS IV (*p* = 0.0094). Motor function improvements were paralleled by a numerical increase in activities of daily living scores (MDS-UPDRS II) and improvement in long-term mobility (PDQ-39 mobility subscale), suggesting potential benefits in daily functioning and perceived mobility.

**Conclusion:**

The value of our data is limited by its retrospective design and small sample size. However, the data suggest that a proper day-clinic setting enables the successful implementation of subcutaneous LDp/CDp therapy with improvement of motor functions and reduction of side effects. It also ensures the necessary intensive medical support and offers comprehensive device-related and psychosocial guidance for both patients and caregivers.

## 1 Introduction

Parkinson’s disease is a neurodegenerative disorder characterized by the progressive loss of dopaminergic neurons in the substantia nigra (Kalia and Lang, 2015). This results in an increasing need for exogenously supplied levodopa (Schapira et al., 2009). In advanced Parkinson’s disease (aPD) motor and non-motor symptom fluctuations occur despite optimization of oral dopaminergic medication (Antonini et al., 2018). Several factors contribute to these fluctuations. These include ongoing neurodegeneration and pharmacological issues, such as fluctuating levodopa plasma levels and pulsatile stimulation of striatal dopamine receptors. Additionally, plastic changes in the striatum and sensitization of dopamine receptors play a role. The situation is further complicated by gastrointestinal dysfunction, which affects drug absorption (Bestetti et al., 2017; Nutt et al., 2000). To address these challenges, various infusion therapies for continuous dopaminergic delivery have been developed. These therapies aim to bypass irregular gastrointestinal emptying, including the use of the subcutaneous apomorphine pump and the intrajejunal administration of levodopa/carbidopa intestinal gel via a percutaneous enteral jejunostomy (Antonini et al., 2023). Recently, subcutaneous levodopa in the form of foslevodopa/foscarbidopa (LDp/CDp) has been introduced as a new therapeutic option for patients with aPD. LDp/CDp is a water-soluble drug precursor that achieves a stable pharmacokinetic profile over 72 h through enzymatic conversion to levodopa/carbidopa (Rosebraugh et al., 2021a,b, 2022). In phase 3 studies, a reduction in OFF time and an increase in ON time without dyskinesia was achieved (Aldred et al., 2023; Soileau et al., 2022). Furthermore, it has been shown to improve non-motor symptoms, including sleep and quality of life (Aldred et al., 2023; Chaudhuri et al., 2024). In clinical trials, LDp/CDp-pump has been implemented in an outpatient setting under study conditions (Aldred et al., 2023; Fung et al., 2024; Soileau et al., 2022). However, initiating pump therapy in daily clinical practice may be challenging in outpatient settings due to limited medical and time resources. In contrast, inpatient settings provide comprehensive care but may not reflect the patients’ typical living conditions, potentially overlooking individual medical needs. Previously, we and others have shown that the concept of a Parkinson’s day-clinic proved to be a suitable treatment setting for patients with aPD at the border of in- and outpatient care, providing intensive medical care under everyday conditions (Fründt et al., 2018, 2020; Krause et al., 2022). In line with this, the recently published consensus guideline on Parkinson’s disease by the German Society for Neurology suggested PD patients with medical pumps as eligible patients who likely profit from treatment in a Parkinson’s day-clinic (Höglinger et al., 2024; Hopfner et al., 2024). However, real-world data on LDp/CDp therapy implementation in such day-clinic settings are currently lacking.

In this study, we evaluated the implementation of subcutaneous LDp/CDp treatment in patients with aPD in the therapeutic setting of the Hamburg Parkinson’s day-clinic (HPDC) (Fründt et al., 2018).

## 2 Patients and methods

### 2.1 Patients

We report data of all patients with aPD who were admitted for initiation of LDp/CDp continuous subcutaneous infusion treatment to the HPDC from January until December 2024.

### 2.2 Methods

Treatment in the HPDC includes a defined number of full-day visits (usually 5) of 7–8 h/day within three consecutive weeks (Fründt et al., 2018). Parameters regarding motor and non-motor symptoms were recorded before LDp/CDp therapy initiation (baseline, T0) and twice in the follow-up, once at discharge from HPDC (T1 after 3 weeks), and furthermore 6 weeks later after ambulatory stay (T2 after 9 weeks from baseline). For T2 follow-up, patients were readmitted for 1 day to the HPDC for clinical assessment and therapy optimization under long-term LDp/CDp treatment.

Six day-clinic visits were initially scheduled for these patients within the first 3 weeks, with the option of extending the number of visits if medically necessary. The initial pump device instrumentation was carried out by the medical staff on the day of admission (T0). The second visit 1 day later served as a short-term follow-up to assess the acute therapeutic effect and, if needed, adjust the dosage. The remaining 4 visits over the following 2.5 weeks focused on training patients and their caregivers in the correct and hygienic use of the pump, as well as on further fine-tuning the medication dosage. The scheduled intervals between visits were: 1 day between the first two visits, 2 days between the second and third visits, 6 days between the third and fourth visits, 5 days between the fourth and fifth visits, and 3 days between the fifth and sixth visits. Patients adjusted to LDp/CDp subcutaneous treatment participated in the integrated day-clinic program previously described by us (Fründt et al., 2018). This program was designed to meet their individual needs in terms of individual and group therapy sessions accompanied and managed by an interdisciplinary medical and nursing team as well as therapists from the fields of speech therapy, occupational therapy, physiotherapy and consultants from the fields of social medicine and nutritional medicine. Due to previously described interactions between long-term levodopa treatment and Vitamin B12 (Ceravolo et al., 2013; Mancini et al., 2014), blood serum controls were performed on the day of admission and 9 weeks after LDp/CDp therapy initiation with special regard to Vitamin B 12 levels.

As outcome parameters we included the routinely assessed outcome parameters Unified Parkinson’s Disease Ranking Scale (MDS-UPDRS) part II, III (measured in the ON-state but not based on a formal Levodopa challenge test) and IV (Goetz et al., 2008) reflecting the patient’s motor experiences of daily living (II), the clinical motor examination (III) and motor complications (IV). The MDS-UPDRS IV reflects (i) the waking time spent with dyskinesias, (ii) the functional impact of dyskinesias, (iii) painful off state dystonia, (iv) the time spent in the off state, (v) the functional impact of fluctuations and (vi) complexity of motor fluctuations. The Parkinson’s Disease Questionnaire (PDQ-39) was used for the assessment of quality of life (Jenkinson et al., 1997). All scores and questionnaires were collected before the start of pump therapy (T0), after 3 weeks (T1) and after a total of 9 weeks (T2). All scores were recorded in the clinical ON-state. Patients who did not or incompletely filled out the questionnaires were excluded from pairwise comparative analyses (MDS-UPDRS II: *n* = 3 at T1, *n* = 1 at T2, MDS-UPDRS IV: *n* = 1 at T1, PDQ-39: *n* = 2 at T1, *n* = 1 at T2). Furthermore, we assessed the dynamics of levodopa equivalent daily dosage (LEDD) and vitamin B blood levels and evaluated adverse effects, their treatment and therapy discontinuation rates.

We calculated LEDD according to published guidelines (Tomlinson et al., 2010) as the amount of oral levodopa (retarded and non-retarded) and, if applicable, of co-medication of dopamine agonists (DA) and catechol-*O*-methyltransferase (COMT) and/or monoamine oxidase- (MAO) B inhibitors daily dosage replaced through LDp/CDp and/or continued after LDp/CDp treatment initiation. The hourly basic running rate was calculated according to the manufacturer’s instructions considering the higher bioavailability of LDp/CDp compared to LD (8%) and the molecular weight ratio of LDp/CDp versus levodopa (1.41) (PatientenInfo-Service, 2023). In general, there are three running rates for the different needs and times of the day. The basic running rate covers the patient’s time awake assumed to be 16 h. The lower running rate for assumed 8 h of sleep was generally 80% of the basic running rate. On demand, the higher running rate was used for exercise or in OFF-periods, which was usually 0.02 ml/h higher (equivalent to 34 mg levodopa) than the basic running rate. In the follow-up, adjustments of LDp/CDp dosage and concomitant oral and/or transdermal medication (DA, COMT inhibitors, MAO-B inhibitors) were done as clinically appropriate.

### 2.3 Adverse events

At each visit, patients and their caregivers were systematically asked about potential side effects. A neurological examination was conducted, along with inspection of the abdominal skin. In cases where inflammatory skin reactions were suspected, C-reactive protein (CRP) levels and white blood cell counts were assessed, and ultrasound imaging was performed when clinically indicated.

### 2.4 Statistical analysis

All data are presented as mean ± standard deviation (SD), median ± interquartile range (IQR) or as numbers (percentage) of scores, as indicated. Because of the small cohort size, statistical analyses were performed by non-parametric Friedman test with pairwise comparison and Dunn’s correction for paired data for repeated measures across T0, T1 and T2. For pairwise comparisons, non-parametric Wilcoxon signed-rank test was performed. For unpaired comparisons, non-parametric Mann-Whitney test was applied. *P* < 0.05 indicates statistical significance. Statistical analyses and figures were performed with GraphPad Prism (version 9.5.1 for Windows; GraphPad Software, LLC).

### 2.5 Ethics

The study is a retrospective analysis of clinical data conducted in accordance with the Declaration of Helsinki. According to the local ethics committee of Hamburg there are no objections to publish this kind of data (reference number PV5799, WF-028/18).

## 3 Results

### 3.1 Patients

24 patients with advanced Parkinson’s disease (aPD) (12 males, 5 with deep brain stimulation) experiencing insufficient symptom control (e.g., motor fluctuations, dyskinesias) under their prior therapy, were admitted to the HPDC for initiation and adjustment of LDp/CDp treatment and enrolled in this study. A total of 7/24 patients (4 males) discontinued LDp/CDp treatment because of adverse treatment-related effects (*n* = 4) or averseness to the pump device (*n* = 3) and were excluded from data analysis.

Characteristics of all patients before LDp/CDp therapy initiation are summarized in Table 1. Briefly, mean age was 72.0 ± 9.2 years; disease duration was 13.7 ± 5.4 years; mean Hoehn & Yahr stage was 2.4 ± 0.5. Mean Montreal Cognitive Assessment (MoCA) score was 24.3 ± 3.5.

**TABLE 1:** Patient characteristics.

Patient	sex	Age, y	Disease duration, y	Hoehn & Yahr	MoCA (ON)	DBS	Number of visits	Concomitant medication		Adverse events	Reasons for therapy discontinuation
								Prior LDp/CDp	T2		
1	M	65	20	2,5	26	No	–	COMT, MAO-B	na	Cellulitis	Cellulitis
2	F	80	16	4	18	No	7	COMT, MAO-B	–	Cellulitis	
3	F	77	17	3	26	No	–	DA	na		Averseness to device
4	M	66	12	3	30	Yes	8	DA, amantadin	DA, amantadin	Cellulitis	
5	M	69	16	2	18	No	–	COMT	na	Abscess	Abscess
6	F	89	5	2,5	24	No	8	COMT, MAO-B	–		
7	M	71	11	2,5	24	No	–	COMT, MAO-B	na	Neuropsychiatric (hallucinations, disorientation)	Hallucinations
8	F	61	10	2	20	No	7	COMT, DA, MAO-B	MAO-B		
9	F	54	13	2	24	No	9	COMT	–	Abscess	
10	F	65	16	2	21	Yes	7	COMT	–		
11	M	67	11	2	26	No	8	COMT, DA, MAO-B	COMT, DA (reduced)	Hallucinations	
12	M	77	21	3	22	No	7	COMT, DA	COMT		
13	F	80	23	2	27	Yes	9	DA	DA	Abscess	
14	M	88	18	3	–	No	9	MAO-B, amantadin	–	Hallucinations, delusions	
15	M	82	9	2	27	No	–	–	na		Averseness to device
16	F	80	15	2	19	No	–	–	na		Averseness to device
17	M	81	18	2	23	No	7	DA, MAO-B	DA, MAO-B		
18	F	72	10	2	23	No	8	COMT, DA, MAO-B	COMT		
19	M	70	4	3	27	No	8	MAO-B	MAO-B		
20	F	68	8	2	25	No	–	COMT	na	OFF dyskinesias	OFF dyskinesias
21	F	71	8	2	25	No	8	DA, MAO-B	COMT, DA, MAO-B		
22	M	62	16	2	30	Yes	7	COMT, DA	DA (reduced)		
23	M	77	23	2	24	No	8	COMT, DA, MAO-B	COMT, DA (reduced), MAO-B		
24	F	57	9	2	30	Yes	7	–	–		
**Mean ± SD**		72.0 ± 9.2	13.7 ± 5.4	2.4 ± 0.5	24.3 ± 3.5		7.8 ± 0.8				

Characteristics of all patients included (*n* = 24). Values are given as mean (SD). Y, years. Disease duration is calculated from symptom onset to initial pump device instrumentation. MoCA was assessed in MED and STIM ON. DBS, deep brain stimulation. Number of visits includes the total number of visits in the Parkinson’s day-clinic during the 9-week observation period. COMT, COMT-inhibitors (entacapone, opicapone); MAO-B, MAO-B-inhibitors (rasagiline, safinamide); DA, dopamine agonists (ropinirole, pramipexole, rotigotine, piribedil).

Six day-clinic visits were initially scheduled within the first 3 weeks, with the option to extend the number of visits if medically necessary. On average, patients had 7.8 ± 0.75 visits in the day-clinic during the 9-week study observation period. Filling of the pump syringe and replacement of the pump infusion set were demonstrated and supervised as individually needed. Repeated instructions to patients and their caregivers were necessary to ensure a successful transition to independent pump use in the patients’ daily lives.

### 3.2 Pharmacological and laboratory aspects

**FIGURE 1:**
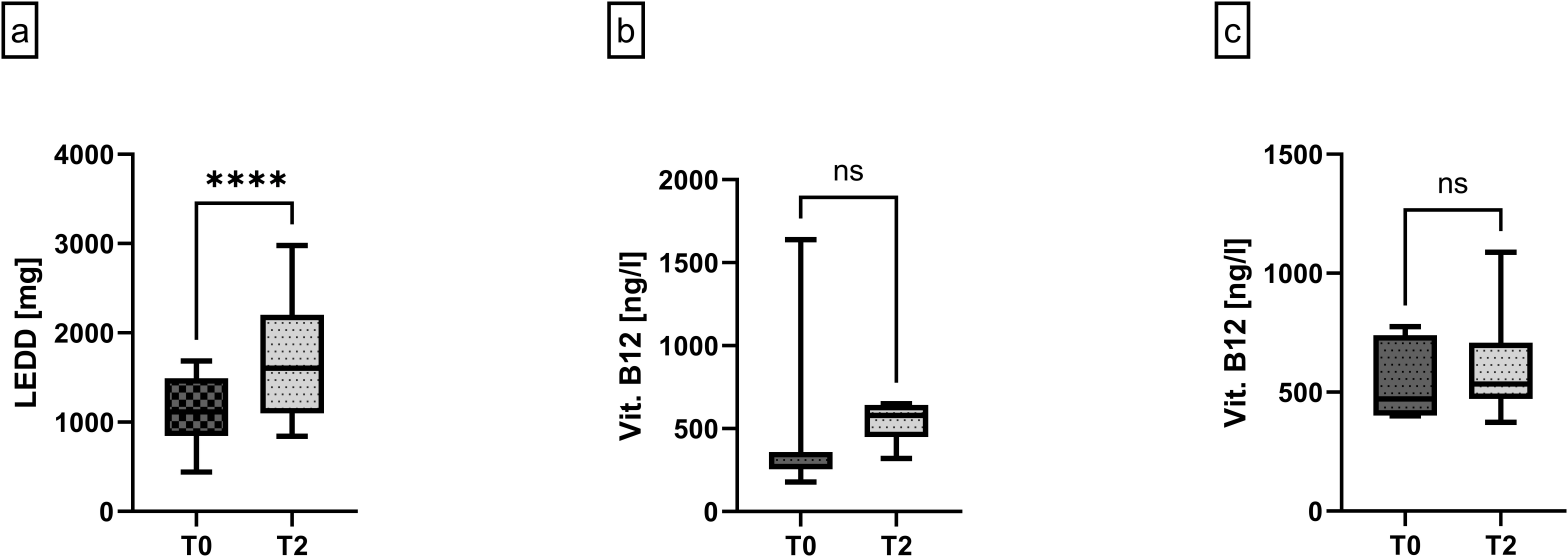
**(a)** Levodopa equivalent daily dosage (LEDD) [mg] at baseline (T0) and after 9 weeks under LDp/CDp treatment (T2), representing the sum of all (fos-)levodopa-containing and dopaminergic concomitant medication at the respective timepoint. *n* = 17/24 at T0 and T2. **(b)** Vitamin B12 serum levels [ng/I] in patients with Vitamin B12 supplementation during the 9-week observation period. *n* = 7/13 per timepoint. **(c)** Vitamin B12 serum levels [ng/I] in patients with no Vitamin B12 supplementation during the 9-week observation period. *n* = 6/13 per timepoint. 4 patients without Vitamin B12 level control at T2. All data are presented as median ± interquartile range, and minimum/maximum, non-parametric Wilcoxon test, ****p < 0.0001, ns, not significant.

The LEDD significantly increased by 55.07% 9 weeks after initiation of LDp/CDp treatment [T0 1115 mg (843.5–1491), T2 1605 mg (1099–2203, *p* < 0.0001)]. (Figure 1a) Monotherapy with LDp/CDp was considered the most appropriate treatment in approximately one third of patients (35%, *n* = 6). Before starting treatment, 10 patients were receiving concomitant therapy with a dopamine agonist. This therapy was reduced or discontinued in six of these cases. COMT inhibitors were withdrawn in four of ten patients, while MAO-B inhibitors were discontinued in six of eleven patients. A new COMT inhibitor was started in one patient. Detailed information on concomitant medications is provided in Table 1.

Seven out of the 17 patients completing the 9-week periods who had Vitamin B12 levels below 400 ng/l at T0 were substituted with Vitamin B12 either 1000 μg *per os* per day for 3 months (*n* = 5) or five times 1000 μg intramuscular (*n* = 2) within the study period of 9 weeks. Both groups with and without vitamin B12 supplementation had a comparable Vitamin B12 level at T2 compared to T0 [supplemented group median 580 ng/l (449–642) at T2 and median 344 ng/l (255–359) at T0, unsupplemented group median 534 ng/l (471.3–707.8) at T2 and median 472 ng/l (401.8–739.8) at T0] (Figures 1b, c).

### 3.3 Adverse events

Inflammatory skin reactions at the treatment site were observed in 6/24 patients (25%) during the 9-week observation period. Of these, four cases were diagnosed as cellulitis and two as abscesses according to the clinical signs – redness, warming, swelling and pain – as well as sonographic display of subcutaneous encapsulated accumulation of fluid when appropriate. All reactions were associated with a slight to moderate elevation in C-reactive protein (CRP) levels (up to 27 mg/L), without accompanying leukocytosis. Systemic signs of inflammation, such as fever or shivering, were not observed. All affected patients received antibiotic treatment: five were treated with amoxicillin/clavulanic acid (875/125 mg) for 5–7 days, and one with clindamycin (600 mg for 5–7 days) due to penicillin allergy. The two patients with abscess formation additionally had to undergo surgical abscess cleavage.

Notably, 5 of the in total 6 patients with skin reactions occurred in our first 10 patients. As a result, adjustments were made to the handling of the infusion system and the infusion site. While the infusion needle was initially changed every 72 h in accordance with the manufacturer’s instructions, we reduced this interval to 24 h. Furthermore, smoothing out the previous abdominal infusion site with a massage ball and leaving the previous infusion needle in place for a further 60 min after discontinuation of the system were done with beneficial effect.

Following these adjustments, the incidence of inflammatory site reactions related to LDp/CDp treatment was reduced, with only 1 of the remaining 14 patients (7.1%) developing a subcutaneous abscess.

2 patients discontinued therapy due to inflammatory skin reactions.

Psychotic symptoms requiring treatment occurred in a total of three patients. Two male patients with a prior history of medication-induced hallucinations experienced recurrent hallucinations during LDp/CDp therapy. One further patient suffered from mild hallucinations already at treatment initiation which became more severe. For one of these patients, this resulted in discontinuation of therapy. The other two patients were treated temporally with a reduction in LDp/CDp dosage and clozapine 12.5 mg/d and 6.25 mg/d, respectively. None of the cases required hospitalization.

Among the remaining 15 patients who completed the 9-week observation period without developing psychotic symptoms, 60% (9/15) also had a documented history of delirium or medication-induced hallucinations and delusions. There were no significant differences in baseline MoCA score [patients who developed hallucinations median 25 (24–26), patients without hallucinations median 24 (22–27)], MDS-UPDRS III [patients with hallucinations median 30 (20–67), patients without hallucinations median 30 (28–42) of] or age [patients with hallucinations median 71 (67–88), patients without hallucinations median 71 (62–80)] between patients who developed psychosis and those who did not under LDp/CDp therapy. The initial LEDD tended to be higher in patients who developed hallucinations [with hallucinations median 1499 mg (791–1866), without hallucinations median 1115 mg (861–1490)] (Supplementary Figure 1).

One patient (female) developed paradoxical off-dyskinesias despite increase of LDp/CDp dosage which were reversed after oral intake of soluble levodopa.

### 3.4 LDp/CDp improved motor functions and reduced dyskinesias and motor fluctuations

**FIGURE 2:**
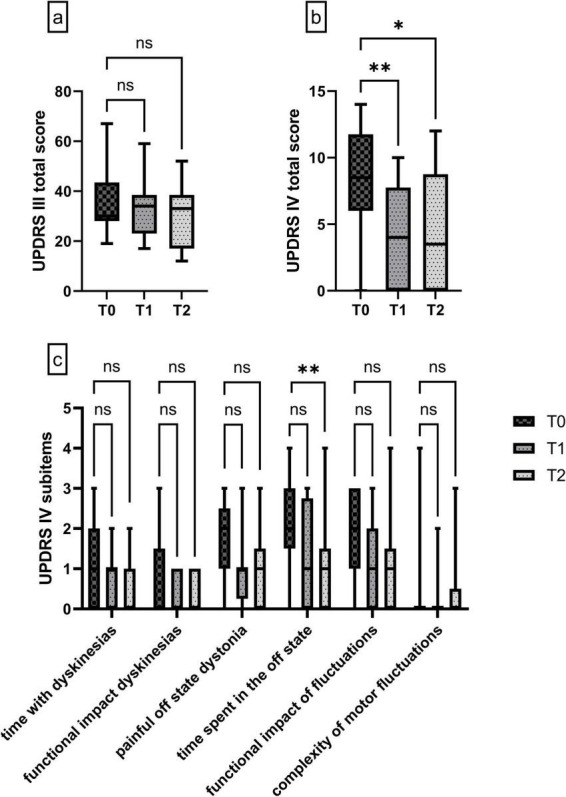
**(a)** UPDRS Ill total score at T0, T1 and T2 under LDp/CDp (*n* = 17). **(b)** UPDRS IV total score at T0, T1 and T2 under LDp/CDp (*n* = 16). **(c)** Subitems of the UPDRS IV at T0, T1 and T2 under LDp/CDp (*n* = 16). Data are presented as median ± interquartile range, and minimum/maximum. Non-parametric Friedman test **(a–c)** with Dunn’s multiple comparison analysis. */**p < 0.05, ns, not significant.

Median ON-state MDS-UPDRS III score of the 17/24 patients under continuing LDp/CDp therapy remained stable at T1 and T2 [T0 median 30 (28–43.5), T1 median 34 (23–38.5), T2 median 33 (17–38.5)] (Figure 2a).

Median MDS-UPDRS IV score improved significantly by 53% from T0 to T1 [T0 median 8.5 (6–11.75), T1 median 4 (0–7.75), *p* = 0.0094], indicating reduction of dyskinesias, motor fluctuations, OFF phases and the subsequent impairments experienced by patients. This level of reduction of motor complications could be basically maintained at T2 9 weeks later with a median score of 3.5 [(0–8.75), *p* = 0.0267)] (Figure 2b).

Analysis of subcategories of the MDS-UPDRS IV revealed a possible reduction of the time spent with dyskinesias by 33% from median 1.5 (0–2) at T0 to 1 (0–1) at T1 and by 100% to median 0 (0–1) at T2. The functional impact of dyskinesias remained unchanged with a median score of 0 at all time points [T0 median 0 (0–1.75), T1 median 0 (0–1), T2 median 0 (0–1)]. Frequencies of painful off-states [T0 median 2 (1–2.75), T1 median 1 (0.25–1), T2 median 1 (0–1.75)] as well as the time spent in off states [T0 median 2 (1.25–3), T1 median 1 (0–2.75), T2 median 0.5 (0–1)] were both possibly reduced in the short-term at T1 by 50%, respectively (*p* > 0.05), as well as in the long-term treatment at T2 by 50% (painful off-states, *p* > 0.05 for T0 vs. T2) and 75% (time spent in the off state, *p* = 0.0094 for T0 vs. T2). The analysis of the functional impact of motor fluctuations revealed a possible reduction of 50% from median 2 (1–2.75) at T0 to median 1 (0–2) at T1 and median 0.5 (0–1.75) at T2. The complexity of motor fluctuations showed neither a statistical significance nor a clear tendency (Figure 2c).

**FIGURE 3:**
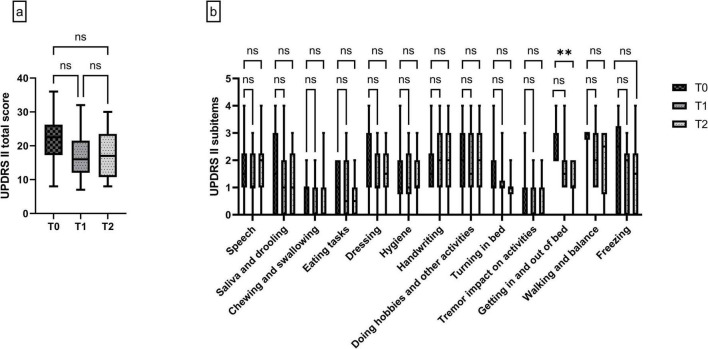
**(a)** Total score of UPDRS II “motor experiences of daily living” at T0, T1 and T2. **(b)** Subitems of UPDRS II at T0, T1 and T2. Data are presented as median ± interquartile range, and minimum/maximum. *n* = 14 at all timepoints. Non-parametric Friedman test with Dunn’s multiple comparison analysis, **p = 0.007, ns, not significant.

The objective improvement of motor functions as well as the possible reduction of motor complications were reflected in the subjective assessment of motor functions within the MDS-UPDRS II mirroring the patient’s motor experiences of daily living. Longitudinal analysis of the total MDS-UPDRS II score showed a possible reduction by 29% for the short-term assessment from median 22.5 (17.25–26.25) at T0 to median 16 (12–21.5) at T1 what could basically be maintained at T2 with a median score of 17 (10.75–23.5) (Figure 3a).

Analysis of the subcategories of the MDS-UPDRS II revealed a numerical amelioration regarding “chewing and swallowing” from median 1 (0–1) at T0 to median 0 (0–1) at T1 and T2. No improvement was observed in the categories “turning in bed” [T0: median 1 (1–2), T1 and T2: median 1 (1–1)] and for “tremor impact on activities” [T0: median 0 (0–1), T1 and T2: median 0 (0–1)0]. Significant long-term improvement was observed in the item “getting in and out of bed” by 50% [T0: median 2 (2–3), T1 median 1 (1–2) and T2 median 1 (1–2), *p* = 0.0070 for T0 vs. T2]. “Walking and balance” possibly improved by 33% at both time points compared to T0 [T0 median 3 (3–3), T1 and T2 median 2 (1–3)] and “freezing” with approximately 66% at T2 compared to T0 [T0 median 3 (0–4), T1 median 2 (0–3) and T2 median 1 (0–2)] (Figure 3b).

### 3.5 Improved motor functions reflect improved quality of life

**FIGURE 4:**
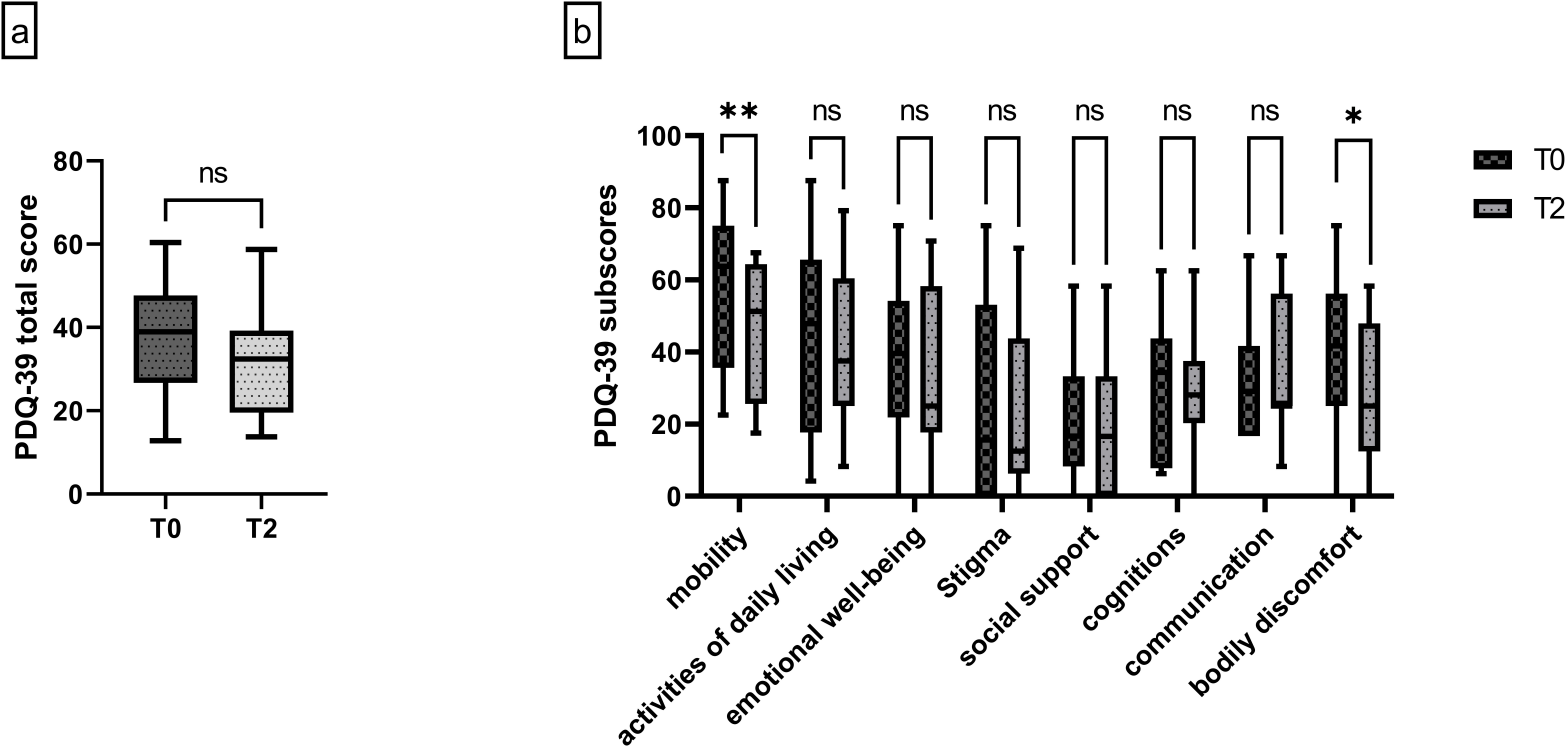
**(a)** PDQ-39 total score at T0 and T2 under LDp/CDp. **(b)** Subscores of the PDQ-39 at T0 and T2. *n* = 16 at both timepoints. Data are presented as median ± interquartile range, and minimum/maximum. Non-parametric Wilcoxon Test **(a,b)**. **p = 0.0015, *p = 0.0127, ns, not significant.

The total PDQ-39 possibly improved in the long-term assessment at T2 by 16.65% compared to baseline [T0 median 38.93 (26.69–47.70), T2 median 32.45 (19.53–39.26)] (Figure 4a).

Sub-analysis of the individual PDQ-39 items revealed a significant improvement in the category “mobility” by a reduction of 19.6% at T2 compared to baseline [T0 median 63.75 (35.63–75), T2 median 51.25 (25.63–64.38), *p* = 0.015]. This indicates improved daily mobility due to LDp/CDp treatment to be a main aspect for improved quality of life. Bodily discomfort also improved significantly by 40% [T0 median 41.67 (25–56.25), T2 median 25 (12.5–47.92), *p* = 0.0127].

Furthermore, other items numerically improved under LDp/CDp treatment, such as activities of daily living [T0 median 47.92 (17.71–65.63), T2 median 37.5 (25–60.42)] and “emotional well-being” [T0 median 39.58 (21.87–54.17) and T2 median 25 (17.71–58.33)] (Figure 4b).

A comprehensive summary of the outcome parameters is given in Table 2.

**TABLE 2:** Outcome parameters.

Item	T0	T1	T2	*P*-value (T1 vs. T0; T2 vs. TO)
LEDD [mg]	1115	–	1605	<0.0001 (T2 vs. T0)
** Vit B12 level [ng/l]**				
With substitution	344	–	580	0.2969 (T2 vs. T0)
Without substitution	472	–	534	0.4375 (T2 vs. T0)
** MDS-UPDRS II**				
Total score	22.5 (17.25–26.25)	16 (12–21.5)	17 (10.75–23.5)	0.3718; 0.1452
Speech	2 (1–2)	1 (1–2)	2 (1–2)	>0.99; >0.99
Saliva and drooling	2 (0–3)	1 (0–2)	2 (1–2)	0.7226; >0.99
Chewing and swallowing	1 (0–1)	0 (0–1)	0 (0–1)	0.4025; 0.2883
Eating tasks	1 (0–2)	1 (0–2)	1 (0–1)	>0.99; >0.99
Dressing	2 (1–3)	1 (1–2)	2 (1–3)	0.1657; 0.2414
Hygiene	1 (1–2)	1 (1–2)	1 (1–2)	>0.99; >0.99
Handwriting	2 (1–2)	2 (1–3)	2 (1–3)	0.8226; >0.99
Doing hobbies	2 (1–3)	2 (1–3)	2 (1–3)	0.5466; 0.8226
Turning in bed	1 (1–2)	1 (1–1)	1 (1–1)	>0.99; 0.4025
Tremor	0 (0–1)	0 (0–1)	0 (0–1)	0.6306; >0.99
Getting out of bed	2 (2–3)	1 (1–2)	1 (1–2)	0.0892; 0.007
Walking and balance	3 (3–3)	2 (1–3)	2 (1–3)	>0.99; 0.4707
Freezing	3 (0–4)	2 (0–3)	1 (0–2)	0.2414; 0.0892
MDS-UPDRS III (ON)	30 (28–43.5)	34 (23–38.5)	33 (17–38.5)	0.2065; 0.0516
** MDS-UPDRS IV**				
Total score	8.5 (6–11.75)	4 (0–7.75)	3.5 (0–8.75)	0.0094; 0.0267
Time with dyskinesias	1.5 (0–2)	1 (0–1)	0 (0–1)	0.2659; 0.0543
Impact of dyskinesias	0 (0–1.75)	0 (0–1)	0 (0–1)	0.4318; 0.5011
Time in OFF state	2 (1.25–3)	1 (0–2.75)	0.5 (0–1)	0.1037; 0.0094
Impact of fluctuations	2 (1–2.75)	1 (0–2)	0.5 (0–1.75)	0.1269; 0.1269
Complexity of fluctuations	0 (0–0)	0 (0–0)	0 (0–0.75)	>0.99; >0.99
Painful OFF dystonia	2 (1–2.75)	1 (0.25–1)	1 (0–1.75)	0.1037; 0.1542
** PDQ-39**				
Total score	38.93 (26.69–47.7)	–	32.45 (19.53–39.26)	0.0507 (T2 vs. T0)
Mobility	63.75 (35.63–75)	–	51.25 (25.63–64.38)	0.0015 (T2 vs. T0)
Activities of daily living	47.92 (17.71–65.63)	–	37.5 (25–60.42)	0.7719 (T2 vs. T0)
Emotional well-being	39.58 (21.87–54.17)	–	25 (17.71–58.33)	0.2333 (T2 vs. T0)
Stigma	15.63 (0–53.13)	–	12.5 (6.25–43.75)	0.5098 (T2 vs. T0)
Social support	16.67 (8.33–33.33)	–	16.67 (0–33.33)	0.5049 (T2 vs. T0)
Cognition	34.38 (7.813–43.75)	–	28.13 (20.31–37.5)	0.5643 (T2 vs. T0)
Communication	29.17 (16.67–41.67)	–	25 (25–56.25)	0.3594 (T2 vs. T0)
Bodily discomfort	41.67 (25–56.25)	–	25 (12.5–47.92)	0.0127 (T2 vs. T0)

Overview of all outcome parameters at time points T0 (before LDp/CDp therapy), T1 (after 3 weeks), and T2 (after 9 weeks).

## 4 Discussion

Our data suggest that the Hamburg Parkinson-day clinic (Fründt et al., 2018; Krause et al., 2022) is a suitable clinical setting for the successful implementation of subcutaneous LDp/CDp therapy in patients with aPD. In this setting, LDp/CDp treatment initiation was associated with improvements in overall motor fluctuations, as assessed by the MDS-UPDRS IV. These motor improvements may contribute to better performance in daily activities and enhanced quality of life, particularly regarding mobility and bodily discomfort. Additionally, adjustments to device handling appeared to reduce skin reactions and may have supported better therapy adherence.

Both phase III LDp/CDp trials (Aldred et al., 2023; Soileau et al., 2022) were performed in an outpatient setting under study conditions. Nevertheless, the optimal clinical setting for LDp/CDp therapy initiation remains unclear. We found that the adjustment of subcutaneous LDp/CDp infusion treatment requires short-term follow-up visits optimally within daily and weekly periods which would be challenging in outpatient settings. In contrast, while inpatient neurological units allow for closer monitoring, they fail to replicate the patient’s typical home and work environments. Consequently, treatment outcomes observed at discharge from inpatient settings often do not persist once patients return to their everyday lives.

Our findings support that a specialized day clinic is an appropriate setting for beginning complex treatments, such as subcutaneous LDp/CDp therapy. The HPDC concept enables close monitoring of treatment effects and ensures proper patient training in device handling. This approach benefits from structured medical support, including sufficient staffing, extended observation periods during daily visits, and frequent clinician-patient interactions. The initiation and titration of LDp/CDp therapy require intensive supervision, which can be effectively provided in a day clinic. This level of support may contribute to a reduction of discontinuation rates, which were as high as 35% and 43.9% within 12 and 52 weeks, respectively, in pivotal LDp/CDp trials (Aldred et al., 2023; Soileau et al., 2022).

Among the first 10 patients treated, we observed a therapy discontinuation rate of 40%, comparable to those reported in previous studies (Aldred et al., 2023; Soileau et al., 2022). The primary reason for discontinuation was inflammatory skin reactions at the abdominal infusion sites, occurring in 50% of patients. In response, we implemented several modifications to device management, hygiene protocols, and patient counseling. These included changing infusion needles and accessories every 24–48 h, optimizing the timing of needle removal, massaging previous infusion sites, and providing intensified support to patients and caregivers. These interventions led to a reduction in the therapy discontinuation rate to 21.4% and a decrease in inflammatory skin reactions to 7.1% among the subsequent 14 patients.

Due to availability issues, it was not possible to demonstrate the pump to the patients included in this study prior to the start of therapy. Consequently, three patients discontinued therapy due to their inability to cope with the pump’s size and handling. This issue should be resolved as demo pumps become more widely available.

Our approach to initiating LDp/DCp therapy differed in some aspects from previous protocols (Aldred et al., 2023; Chaudhuri et al., 2024; Soileau et al., 2022). While in both studies COMT-inhibitors, but not MAO-B inhibitors, were discontinued prior to LDp/CDp treatment initiation, we followed this concept only in the first patients but then kept concomitant COMT- and/or MAO-treatment to save LDp/CDp dosage with good tolerability. We speculate that a lower LDp/CDp infusion rate may be beneficial in terms of better drug resorption and fewer skin reactions.

In our study, 35% of patients finally were treated with LDp/CDp monotherapy, slightly exceeding 25.7% and 20.1% reported in the 12- and 52-weeks trials, respectively (Aldred et al., 2023; Soileau et al., 2022). However, in our opinion reaching monotherapy should not be a primary aim. In patients with high-dose dopamine agonist therapy prior to LDp/CDp therapy, dosage of agonists should only carefully and stepwise be reduced after LDp/CDp treatment initiation to avoid withdrawal syndrome (Yu and Fernandez, 2017). The increase in LEDD of over 50% after 9 weeks can be explained, on the one hand, by the pump running at night, which alone leads to an increase in LEDD of approximately 30% (given a nighttime running rate of 60%–80% of daytime). Additionally, continuous delivery may prevent L-dopa peaks and reduce peak-dose dyskinesia, even at higher total doses. A recently published study reported an LEDD increase of 55% and even 65% respectively, based on already higher baseline values (Brohée et al., 2025). Though, information on concomitant medications was not provided. However, the significantly higher LEDD could be responsible for the high rate of neuropsychiatric symptoms in that cohort. In our cohort, patients who developed hallucinations and LDp/CDp also tended to have a higher LEDD but did not differ in terms of age, cognitive status, or motor function. However, the group with hallucinations was very small.

The therapeutic effects observed in the HPDC after initiation of LDp/CDp treatment are in line with those reported in previous studies (Aldred et al., 2023; Soileau et al., 2022). Overall, motor complications measured using the MDS-UPDRS IV were reduced. The data show an improved mobility in patients, which may be associated with enhanced quality of life. Clinical improvements observed in this study appeared to predominantly affect axial motor domains, whereas fine motor functions, such as handwriting, remained largely unaffected. While the limited sample size and absence of objective fine motor assessments constrain definitive conclusions, one may hypothesize that the reduction of “partial ON” states by LDp/CDp may underlie the observed benefits on axial symptoms. Comparable effects have been reported with intrajejunal levodopa-carbidopa infusion, which has demonstrated efficacy in ameliorating axial motor deficits, particularly gait disturbances, that are typically refractory to oral levodopa therapy. (Chang et al., 2015; Imbalzano et al., 2023) These findings suggest a potential class effect of continuous levodopa delivery systems on axial symptomatology. Further investigations in larger cohorts employing quantitative kinematic assessments are warranted to elucidate whether subcutaneous levodopa delivery exerts similar benefits.

Limitations of our study include the small number of patients, uncontrolled design, and short follow-up period.

## 5 Conclusion

We were able to show that the beneficial effects of LDp/CDp occurred not only within the 3-week day-clinic stay, but were maintained at least up to 9 weeks, eliminating a potential bias due to intensified care during the initial 3-week day-clinic stay.

In summary, LDp/CDp treatment can be implemented successfully within an adequate Parkinson’s day-clinic setting with a reduction in motor complications comparable to study results but eventually even better tolerability with less side effects due to device-related modifications and the advantages of the day-clinic concept.

## Data Availability

The raw data supporting the conclusions of this article will be made available by the authors, without undue reservation.
